# Equitable infrastructure: Achieving resilient systems and restorative justice through policy and research innovation

**DOI:** 10.1093/pnasnexus/pgae157

**Published:** 2024-04-15

**Authors:** Jason P Giovannettone, Gregg P Macey, Amir AghaKouchak, Michele Barbato, William J Capehart, Auroop R Ganguly, Mital Hall, Jennifer F Helgeson, Si Han Li, Teng Wu, Guirong Yan, Farshid Vahedifard

**Affiliations:** Department of Climate and Sustainability, Sisters of Mercy of the Americas, Inc., 8403 Colesville Rd., Silver Spring, MD 20910, USA; Center for Land, Environment, and Natural Resources, School of Law, University of California, Irvine, 401 East Peltason Drive, Irvine, CA 92697, USA; Brooklyn Law School, 250 Joralemon St, Brooklyn, NY 11201, USA; Department of Civil & Environmental Engineering, University of California, Irvine, 4130 Engineering Gateway, Irvine, CA 92697, USA; Department of Earth System Science, University of California, Irvine, 3200 Croul Hall, Irvine, CA 92697, USA; United Nations University Institute for Water, Environment, and Health (UNU-INWEH), 225 East Beaver Creek Rd, Richmond Hill, ON L4B 3P4, Canada; Department of Civil and Environmental Engineering, University of California, Davis, One Shields Avenue, Davis, CA 95616, USA; Department of Civil & Environmental Engineering, South Dakota School of Mines and Technology, 501 E. Saint Joseph St., Rapid City, SD 57701, USA; Department of Civil and Environmental Engineering, Northeastern University, 177 Huntington Avenue, Boston, MA 02115, USA; RE Tech Advisors, Windermere, 1676 International Drive, Suite 600, McLean, VA 22102, USA; Applied Economics Office, Engineering Laboratory, National Institute of Standards and Technology, 100 Bureau Drive, Gaithersburg, MD 20899, USA; Rowan Williams Davies & Irwin Inc., 600 Southgate Drive, Guelph, N1G 4P6, Canada; Department of Civil, Structural and Environmental Engineering, University at Buffalo, 212 Ketter Hall, Buffalo, NY 14260, USA; Department of Civil, Architectural and Environmental Engineering, Missouri University of Science and Technology, 1401 N. Pine Street, Rolla, MO 65409, USA; United Nations University Institute for Water, Environment, and Health (UNU-INWEH), 225 East Beaver Creek Rd, Richmond Hill, ON L4B 3P4, Canada; Department of Civil & Environmental Engineering, Tufts University, 200 College Avenue, Medford, MA 02155, USA

**Keywords:** equitable infrastructure, resilient infrastructure, climate change adaptation, restorative justice, social, environmental, and economic development (SEED) certification

## Abstract

Recent major investments in infrastructure in the United States and globally present a crucial opportunity to embed equity within the heart of resilient infrastructure decision-making. Yet there is a notable absence of frameworks within the engineering and scientific fields for integrating equity into planning, design, and maintenance of infrastructure. Additionally, whole-of-government approaches to infrastructure, including the Justice40 Initiative, mimic elements of process management that support exploitative rather than exploratory innovation. These and other policies risk creating innovation traps that limit analytical and engineering advances necessary to prioritize equity in decision-making, identification and disruption of mechanisms that cause or contribute to inequities, and remediation of historic harms. Here, we propose a three-tiered framework toward equitable and resilient infrastructure through restorative justice, incremental policy innovation, and exploratory research innovation. This framework aims to ensure equitable access and benefits of infrastructure, minimize risk disparities, and embrace restorative justice to repair historical and systemic inequities. We outline incremental policy innovation and exploratory research action items to address and mitigate risk disparities, emphasizing the need for community-engaged research and the development of equity metrics. Among other action items, we recommend a certification system—referred to as Social, Environmental, and Economic Development (SEED)—to train infrastructure engineers and planners and ensure attentiveness to gaps that exist within and dynamically interact across each tier of the proposed framework. Through the framework and proposed actions, we advocate for a transformative vision for equitable infrastructure that emphasizes the interconnectedness of social, environmental, and technical dimensions in infrastructure planning, design, and maintenance.

## Introduction

Infrastructure has been widely recognized as a critical factor in shaping historically underserved and socially vulnerable communities (HUSVCs) (1, 2). More recently, the role of equity in infrastructure development has been gaining traction in the scientific literature (3–5). With the establishment of “loss and damage” funding, adaptation finance (6), and other significant infrastructure investments, we have a unique opportunity to ensure that equity is fully integrated into infrastructure decision-making processes. However, the engineering and scientific communities lack frameworks to incorporate equity into traditional infrastructure engineering—as opposed to funding—practices. We distinguish the goal of “equity” (7) (ensuring that all people have the opportunity to reach their full potential) from “justice” (8, 9) and its many dimensions under the law (e.g. procedural, distributive, recognitional) for purposes of this article, as further described below. Recent whole-of-government approaches to infrastructure, including the Justice40 Initiative (10, 11), mimic elements of process management that support exploitative rather than exploratory innovation (12). Current policies risk creating innovation traps that limit analytical and engineering advances necessary to (i) prioritize equity in infrastructure decision-making, (ii) identify and disrupt mechanisms that cause or contribute to inequities, and (iii) address and remediate historic harms. In response, we propose a framework that promotes the integration of equity beyond existing policy approaches. This requires the inclusion of not only procedural and distributive but also restorative justice measures (13). The framework aims to reduce uncertainties regarding infrastructure vulnerability and address evolving risks associated with nonstationarity in a changing climate (14). This manuscript is focused on equitable infrastructure within the United States (US), acknowledging that concepts of equity can differ substantially across countries and regions. Therefore, the policy examples discussed are primarily tailored to the US context. Further studies are warranted to examine whether they are adaptable to multi-sector initiatives in other regions, such as those under the European Union's Green Deal (15). Given the diversity in policy frameworks and equity considerations globally, future research will explore this potential applicability.

Three prevailing trends make it increasingly challenging to disregard the dynamic interplay between infrastructure and equity. First, a changing climate exacerbates inequities by increasing exposure and sensitivity to extreme weather events, which highlight systemic inequities characterized by inadequate and aging infrastructure in HUSVCs (16). For example, in Jackson, MS (US), severe storms in August 2022 caused floodwaters to overwhelm the city's largest water treatment facility, which operated under marginal conditions with limited redundancy for years. The US Environmental Protection Agency (EPA) initiated a civil rights investigation to determine whether state agencies had caused adverse disparate impacts to Jackson's predominantly Black population through oversight of the city's water system and administration of clean water revolving funds (17).

Second, as urbanization, regional disparities, and underinvestment in rural, unincorporated, and Indigenous infrastructure continue or accelerate, infrastructure inequities limit efforts to accommodate future growth in a sustainable manner (4, 18, 19). The existing infrastructure is ill-equipped to meet current demand requirements and projected usage as well as cope with changing climatic conditions. Such deficiencies make infrastructure increasingly vulnerable to cascading multi-sectoral failures. For example, recent attempts to account for climate change in hazard modeling and flood risk estimates reveal that future risk will disproportionately fall upon Black communities along the Atlantic and Gulf coasts in the US (16). Compound infrastructure hazards in a changing climate represent another example of what social scientists and environmental justice scholars have emphasized for generations, namely that environmental justice community formation cannot be understood solely through a focus on single points of decision and their resulting disparate impacts. Rather, community formation proceeds through a series of public and private policies and practices that create tangible effects over time across multiple spatial and temporal scales (20).

Third, social movements that link infrastructure and equity have significantly increased in recent years, as exemplified by the Dakota Access Pipeline, Union Hill and the People's Tribunal on Natural Gas Infrastructure, and Flint, Michigan (21, 22). In the US, systemic inequities are brought to light through failed assessment, siting, provision, financialization, and infrastructure maintenance. In response, the $1.2 trillion Bipartisan Infrastructure Law (BIL.) (23) and infrastructure provisions of the Inflation Reduction Act (IRA) (24) aim to address infrastructure equity considerations. Measures include grants, loans, and tax credits for infrastructure buildout, innovation, national security, climate adaptation, and nature-based mitigation. However, current allocation processes and thresholds that target investment benefits in “disadvantaged communities,” as defined by geospatial tools such as the Climate and Economic Justice Screening Tool (25, 26), may not adequately consider policy interactions that contribute to underinvestment, burden, and risk of infrastructure failure; they can also lead to adverse, disparate effects if multi-hazard risk in a changing climate is not properly considered. For the purposes of this article, “multi-hazard risk” includes compound as well as cascading risks to infrastructure posed by natural hazards (27).

### Proposed three-tiered framework toward equitable infrastructure

**Figure 1.**
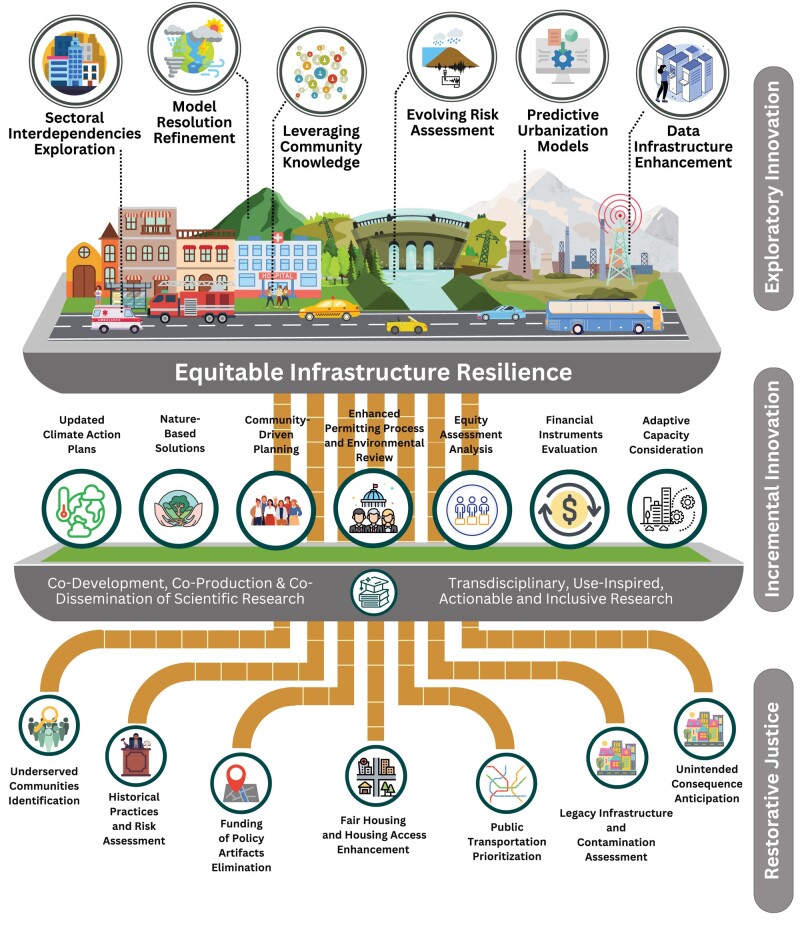
Proposed three-tier equitable infrastructure framework. Conceptual framework toward achieving equitable infrastructure through restorative justice, incremental policy innovation, and exploratory research innovation.

In light of the aforementioned challenges, and in order to address the limits posed by existing policy responses, we define **equitable infrastructure** as the planning, design, and maintenance of infrastructure that (1) minimize risk disparities across HUSVCs and well-resourced communities; (2) prioritize equitable access and benefits for all community members, regardless of socioeconomic status, race, ethnicity, gender, age, disability, or other important indicators of protected status under civil rights laws; and (3) encourage restorative, as opposed to purely procedural or distributive, justice policy design. Equitable infrastructure addresses historical and systemic inequalities (e.g. disparities in the provision of municipal services (28)) by first acknowledging how HUSVCs experience lower risk thresholds and higher adaptive capacity needs for infrastructure components, systems, and system-of-system interactions. To ensure that HUSVCs are not only prioritized via investment (distributive justice) and meaningful involvement (procedural justice) but also given the means to be made whole, we propose a three-tiered framework (illustrated in Figure 1). The framework includes: (1) **restorative justice**, which not only provides an immediate redress to those who have been harmed but also the means for long-term repair and making communities whole (29–31); (2) **incremental policy innovation** in analytical tools that inform planning, regulatory mandates, and financial incentives; and (3) **exploratory innovation** through synergistic advances in science and engineering research that address the impacts of policy implementation on HUSVC formation and susceptibility to hazard nonstationarity. The framework should evolve according to community-engaged and -centered research, adoption and tailoring of equity metrics—including those under development in the US in response to recent executive orders—and monitoring and trend analysis at multiple spatial and temporal scales. While the framework is presented as a progression of policy and scientific developments, much of the innovation will take place through dynamic interactions among two or more elements, within and across tiers. For purposes of the framework, resilience is achieved in part through identifying and addressing unequal adaptive capacities. Resilience, in the context of infrastructure and community planning, refers to the ability of a system to anticipate, prepare for, respond to, and recover from disturbances, shocks, or stresses while maintaining its essential functions and structure (32). It involves the capacity to withstand and absorb impacts and the ability to adapt and transform in the face of changing conditions. Resilience in the infrastructure context includes considerations of both physical and social aspects, ensuring that communities and systems can recover and thrive after disruptions (33, 34). Here we use the term HUSVC to unify the definition of communities that are commonly referred to in the social science and scientific literature as well as in government policy as “underserved,” “disadvantaged,” “low-income and minority,” or “vulnerable.”

#### Tier 1: restorative justice

The proposed framework, illustrated in Figure 1, underscores restorative justice as the cornerstone for achieving equitable infrastructure. Recent policy responses in the US rely on national or state data for the definition and cross-sectional identification of what the federal government refers to as “disadvantaged communities,” (35, 36), thereby recreating the limits of early geospatial research on the distribution of environmental burdens such as locally undesirable land uses (37). For the framework to be effective, it must acknowledge and address the dynamic linkages among infrastructure investment and the policy artifacts—segregation, exclusionary and expulsive zoning, transportation planning, legacy infrastructure and residual contamination, and denial of municipal services among them—that sustain the risk profiles and economic barriers posed by infrastructure systems (38). Strategies such as discontinued funding for practices that include exclusionary zoning, prioritization of auto-centric investment at the expense of transit-oriented development and housing mobility, and infrastructure planning that reinforces segregation (39) can be employed to prevent agencies from locking in or preserving such policy artifacts. By adopting these measures, the framework promotes economic mobility and prevents agencies from perpetuating mechanisms that produce inequitable outcomes. This approach goes beyond codified definitions of environmental justice and agency practices that focus on meaningful involvement of the public (procedural justice (40, 41)) and ensuring equitable distribution of benefits and burdens (distributive justice) to further include (a) understanding the baseline environmental burdens and harms and potential impacts of new projects given project location, size, scale, and interaction effects (recognitional justice) and (b) making communities whole through remediation of legacy pollution, removal of historic barriers, and job, enterprise, and wealth creation via decoupling infrastructure investment from the policy artifacts that perpetuate multi-hazard risk profiles in HUSVCs (restorative justice) (42).

#### Tier 2: incremental policy innovation

With restorative justice as the foundation, we next broaden the current focus of policy to incremental innovation in rules, analytic tools, and financial incentives that are traditionally enacted in isolation. Current analytic tools developed in the US to advance racial equity or environmental justice struggle to link an isolated program, policy, or activity to a disparate impact that threatens a protected class (e.g. race or national origin). To address this limitation, the following environmental review and other policy reforms that enable incremental innovation are necessary. As an initial step, permitting regulations, such as those enacted under the US National Environmental Policy Act, should require consideration of complex risks due to infrastructure disruption and failure under changing climate as “reasonably foreseeable” for purposes of identifying the affected environment as well as indirect and cumulative impacts. Also, under environmental review, the potential to implement feasible mitigation and adaptation measures that stress infrastructure resilience and nature-based solutions should be required under federal and state environmental review laws. Project-based and programmatic reviews should mandate the implementation of feasible mitigation measures to address complex infrastructure disruption and failure risks. To connect restorative justice considerations to multi-hazard risk, mandatory elements in requirements for local and regional general plans and climate action plans should be amended. This includes, for example, consideration of the relationships among infrastructure investment, the lock-in or elimination of policy artifacts, and changing risk profiles for compound events and cascading failures or disruption to infrastructure across historically underserved and well-resourced communities. For example, California law requires local governments to consider wildfire risk and response plans as part of general plan safety elements (SB 1241 (43)), vulnerability assessment, and incorporation of climate risk into hazard mitigation plans (SB 379 (44)), and environmental justice impacts across general plan elements (SB 1000 (45)). Consistency among elements of general, climate action, and sustainability plans (e.g. sustainable community strategies under SB 375 (46))—for purposes of streamlined project-specific greenhouse gas emissions analysis for transportation, energy, and other infrastructure projects—requires amendments that ensure consideration and alignment with plan-identified measures to reverse policy artifacts and address multi-hazard risk. These plans should anticipate future conditions that heighten the risk of compound events and cascading failures or disruptions across multiple sectors and communities while actively working to minimize risk disparities.

Policymakers can also prioritize restorative justice in HUSVCs susceptible to infrastructure disruption and failure through rules, definitions, and financial incentives that are applied *across* sectors and substantive areas of law. For example, all inventories prepared under US Executive Order 14091 (47), where agencies identify community barriers to accessing benefits under existing programs, must include the influence of policy artifacts over infrastructure access, reliability, affordability, and resiliency among their “priority action areas.” Successful implementation would be tracked through mechanisms similar to the Department of Energy's Energy Justice Dashboard to ensure that investments that result in higher vulnerability to multi-system infrastructure disruption do not qualify as benefits. Similarly, legal definitions of “co-benefits” that should be maximized under existing investment programs—such as California's requirement that infrastructure projects funded through cap-and-trade auction proceeds maximize economic, environmental, and public health benefits—should be refined to include reduced vulnerability to multi-system infrastructure disruption. In addition, rules such as the Office of Information and Regulatory Affairs rulemaking review and the duty of federal agencies to affirmatively further fair housing should be applied across historically siloed programs through rulemaking under BIL., IRA, and other statutory regimes to further infrastructure equity in the critical sectors. Laws that set analytical requirements for effective “integration” of local and regional planning with state climate policy should also be amended to require the coordinated governance of infrastructure multi-hazard risk that addresses potential disparate impacts on HUSVCs. In the US, the state of California encourages climate policy integration through a series of measures to align statewide mitigation targets and adaptation goals with multi-pollutant programs, multimodal transportation planning, and local and regional land use, transportation, and sustainable community plans. Broadening statewide definitions of climate policy integration will facilitate coordination across sectors and jurisdictions, which is a key barrier to infrastructure and multi-hazard planning. Finally, federal and state agency rules and guidance should require consideration of the disparate impacts of infrastructure components, systems, and system-of-system interactions within all plans to ensure compliance with civil rights laws such as Title VI of the Civil Rights Act of 1964 (48) and California Government Code § 11135 (49).

Table 1 provides examples of programs in critical sectors that align with these principles, while Table 2 offers examples of incremental policy innovation through cross-sector rules, definitions, and financial incentives. The examples in Table 1 were chosen to cover a range of infrastructure types and sectors to demonstrate the applicability of the framework across diverse contexts. We include examples that highlight various aspects of infrastructure development, including energy, housing, transportation, drinking water, wastewater, hazard planning, disaster response, and public health. It is crucial to identify, inventory, and track all infrastructure programs that receive financial assistance or are managed by government agencies; such actions can enhance compliance with civil rights laws and facilitate multilevel coordination across efforts to manage infrastructure resilience by federal (e.g. coastal levees), state (e.g. highways), and municipal (e.g. stormwater) governments. Moreover, community-specific climate impact metrics (such as the Climate Vulnerability Metric that accompanies California's Scoping Plan update in the US (67)) should be integrated with the location of sensitive populations and incorporate indicators of vulnerability of interconnected infrastructure systems. Such metrics will facilitate compliance monitoring and foster equitable infrastructure development.

**Table 1.** Sector-specific incremental policy innovation.

Sector	Sector-specific Incremental Innovation
Energy	Grid modernization and resilience. Community charging of electric vehicles. Equity-centered electricity outage recovery.
Housing	Community block grants for climate adaptation and resilience. Grants for resilient US Department of Housing and Urban Development-assisted housing. Weatherization and wildfire hardening assistance. Duty to “affirmatively further fair housing” should require consideration of infrastructure siting, location, multi-hazard risk.
Transportation	Protecting transportation infrastructure from extreme weather and other physical hazards. Incorporating green infrastructure.
Drinking water and wastewater	Wastewater access gap reduction. Service line replacement. Coastal restoration. High-quality drinking water access gap reduction. Updating storm design plans to accommodate current and future extreme precipitation.
Hazard planning and disaster response	Hazard mitigation and adaptation. Resilient infrastructure. Community-centered programs. Post-disaster relief. Equity integrated benefit-cost analysis for resilience projects.
Public health	Adaptive capacity for future demands. Integration with climate adaptation to strengthen community resilience.

Examples of actions and innovations to achieve equitable infrastructure by minimizing risk disparities across HUSVCs and well-resourced communities (focus on federal (US) examples).

**Table 2.** Incremental policy innovation through cross-sector rules, mandates, and financial incentives.

Regulatory Instrument	Incremental Policy Innovation
Incentives	Leverage municipal bond markets. Account for climate risk analytics that consider multi-hazard risks when evaluating bond issuers and rating agencies.
Formula funding	Prioritize green infrastructure that mitigates multi-hazard risk (e.g. US Federal Highway Administration's PROTECT Program) and buffers transportation infrastructure from extreme weather.
Climate adaptation and resilience loan guarantees, loan forgiveness, and tax credits	Condition on review of hazard relationships to fossil fuel use. Condition on reduced vulnerability of critical infrastructure in HUSVCs.
Regulations under federal (e.g. National Environmental Policy Act (NEPA)) and state (e.g. California Environmental Quality Act (CEQA)) environmental permitting laws	Revised CEQA regulations (50) should consider increasingly complex risks due to infrastructure disruption and failure under changing climate as “reasonably foreseeable” for purposes of identifying the affected environment, indirect, and cumulative impacts (located at 40 C.F.R. §§ 1502.15, 1502.16, 1508.1); data sources included in screening tools (e.g. (10, 25)) should include complex infrastructure risk via Office of Science and Technology Policy. Executive Order 14096 (51) definition of environmental justice includes “environmental risks” and “hazards” including those related to climate change and structural or systemic barriers; Executive Order 12898 (52) updated via Executive Order 14096 to call for agencies to “identify, analyze, and address” risks and hazards related to climate change, historical inequities “as appropriate and consistent with applicable law”. Scientific integrity of NEPA analyses (40 C.F.R. § 1502.23) should include disparate effects of environmental risks and hazards (53). Cumulative impacts should include capital investments affected by a changing climate (Executive Order 13653 (54)). “Accurate and clear climate change analysis” includes “considering the reasonably foreseeable effects of climate change on infrastructure investments and the resources needed to protect such investments over their lifetime” (53). Alternatives analysis for infrastructure “over the lifetime of the proposed action” should include multi-hazard climate risk (53). NEPA analysis should be integrated with design efforts “at the earliest possible time that would allow for meaningful analysis” (53). Permitting Action Plan calls for improved outcomes of environmental review including community-led mitigation measures (55). Infrastructure resilience and nature-based solutions should be required among options for alternatives analyses under federal and state environmental review laws; project-based and programmatic reviews should mandate the implementation of feasible mitigation measures to address complex infrastructure disruption and failure risks.
Local and regional climate action plans, general plan elements, and sustainability plans	Mandatory elements frameworks should be amended to require consideration of relationships between infrastructure investment and lock-in or preservation of policy artifacts; local climate action and hazard mitigation plans should consider future conditions that pose increasing risk of infrastructure failure or disruption across multiple sectors. California mandatory elements include requirements that local governments consider wildfire risk and response plans as part of general plan safety elements (43), vulnerability assessment and incorporation of climate risk into hazard mitigation plans (44), and environmental justice impacts across general plan elements (45). Consistency requirements for general plan elements and climate action plans for purposes of streamlined greenhouse gas emissions analysis should be amended to include consideration and alignment of infrastructure projects with plan-identified measures to reverse policy artifacts and address multi-hazard risk.
Regulatory impact analysis to determine benefits and costs of significant agency actions (e.g. US Office of Management and Budget's Draft Circular A-4)	Regulatory analysis applied across historically siloed programs under BIL., IRA, and related statutory regimes should consider reduced infrastructure vulnerability to multi-system disruption and failure as “additional benefits” of regulation not accounted for in direct costs and benefits of regulation for purposes of generating superior alternatives (56). Unquantified infrastructure equity indicators should be developed via scenario, screening, or order-of-magnitude analysis for rank ordering. Policy artifacts treated as contextual considerations for purposes of distributional analysis (56). Quantitative analysis of uncertainty should include estimates of probability distributions for environmental damage, harm to human health and safety due to multi-hazard risk (56). Develop unquantified infrastructure equity indicators via scenario, screening, or order-of-magnitude analysis. Revise legal definitions of “co-benefits” that shall be maximized under climate investment programs (e.g. use of cap-and-trade auction proceeds) to include reduced vulnerability to multi-hazard infrastructure risk.
Natural capital accounting	Include value-added investments in nature-based solutions for resilient infrastructure in national economic accounting (e.g. (57)).
State analytical requirements for “integration” of local and regional plans with state climate policy; co-benefits under climate investment programs	Legal requirements for effective “integration” of local and regional planning with state climate policy amended to require coordinated governance of infrastructure multi-hazard risk and potential disparate impacts on vulnerable subpopulations; broadening statewide definitions of climate policy integration will facilitate coordination across sectors and jurisdictions, a key barrier to both infrastructure and multi-hazard planning. California measures to encourage the integration of greenhouse gas emissions reduction and local and regional air quality through multi-pollutant programs (58–60), multimodal transportation planning and state climate goals (61–63), local land use and climate adaptation planning (64, 65), regional transportation and sustainable communities plans with statewide mitigation targets and local housing needs (46). Legal definitions of co-benefits that shall be maximized under existing investment programs should be revised to include reduced vulnerability to multi-system infrastructure disruption.
Federal (e.g. Title VI of the Civil Rights Act of 1964 (48)) and state (e.g. California Government Code § 11135 (49)) agency rules under civil rights laws	Agency rules and guidance under civil rights laws should be amended to require consideration of disparate impacts of infrastructure components, systems, and system-of-system interactions to ensure compliance with civil rights laws. Agency mechanisms to ensure compliance with Title VI and determine if violations should include analysis of interactions of financial assistance with policy artifacts, multi-hazard risk in climate vulnerable communities (e.g. Community Development Block Grant Disaster Recovery program (66)); ongoing data collection, analysis, and consideration to ensure infrastructure investments are consistent with civil rights laws (e.g. Executive Order 14091 (47)).

Actions that promote equitable infrastructure across HUSVCs and well-resourced communities (focus on federal (US) and state/provincial (CA) examples).

#### Tier 3: exploratory research innovation

While the above incremental policy innovations are critical, care should be taken to avoid stifling exploratory research innovation to address the effects of policy implementation on evolving HUSVC risk profiles and disparities. Current design and maintenance procedures tend to focus on weather and climate effects on infrastructure systems at the component level (at the individual infrastructure system level), ignoring system interdependencies (the connection between different infrastructure systems in which one failure can potentially lead to cascading failures) (68). Identifying system-of-systems linkages will require significant exploratory and scenario-based research analysis, including identifying sensitive nodes and impact analysis (27). In addition to accounting for sector interdependencies, regulators should incorporate multi-hazard-resistant designs (69) and codes and standards (70) that address vulnerability to cascading failures within and between sectors (71)—especially under nonstationary climate conditions (i.e. extreme climatic events projected to change in the future) (72)—into the design of infrastructure systems based on life cycle principles (73). Although progress has been made in the design and analysis of infrastructure based on overall performance under nonstationarity (14, 74), significant gaps that create uncertainty and evolving risks remain (Table 3). The examples shown in Table 3 were chosen to reflect the interconnected nature of infrastructure and its impact across multiple sectors. For instance, addressing climate change in transportation planning not only impacts transportation infrastructure but also affects energy policy and public health.

**Table 3.** Exploratory innovation.

Engineering/Scientific Gap	Exploratory Innovation Examples
Lack of attention given to inter- and intra-sectoral interdependence	Advances in methods used to identify and model sectoral interdependencies that contribute to enhanced vulnerability to cascading failures.
Spatial-/temporal-resolution gaps between products derived from global climate models and spatially downscaled products required for local-scale risk assessment	Advances in model resolution and downscaling techniques. Proper consideration of the uncertainties from global climate modeling data. Representation of scientific and engineering data-related interests and expertise within a project.
Limitations in existing methods to understand, characterize, and predict the interplay between two or more natural hazards (i.e. compound events) in a changing climate	Improvement in measurement/projection accuracy of the frequency and magnitude of current and future extreme hazards. Study of the occurrence dependency of individual drivers of a compound event. Increase in emphasis on compound events and their effects on evolving risk and impact intensities under a changing climate.
Lack of approaches to measure progress with respect to equitable infrastructure	Development of metrics in an adaptation communication framework that quantify improvements in equity and evolving risk posed by aging infrastructure under a changing climate. Metrics should address communities characterized by different levels of adaptive capacity, vulnerabilities, and risk thresholds (e.g. “equitable risk” metric).
Limitations in methods to project changes due to an increasing population	Incorporation of changes in urbanization, population, and land use/land cover within predictive models. Improved link to the socioeconomic functions to be met by the built and natural environments.
Data infrastructure and risk communication challenges across engineering and scientific communities of practice	Expertise drawn from multiple scientific and engineering fields and integrated with community and Tribal knowledge to coproduce solutions that address infrastructure vulnerabilities to compound hazards and cascading failures. Public access to dashboard/risk and maps/data visualization tools to benchmark and track vulnerable areas and equitable risk indicators, rather than limiting access to scientists/engineers/planners, to encourage information-based regulation.
Limitations in existing approaches to consider deep uncertainties associated with future climate	Advances in approaches used for climate adaptive planning.

Engineering and scientific gaps that are fundamental to minimizing risk disparities across HUSVCs and well-resourced communities.

To address these gaps, incremental policy innovation grounded in restorative justice should be joined by exploratory innovation through synergistic advances in science and engineering research that address the impacts of policy implementation on HUSVC susceptibility to hazard nonstationarity. The focus of exploratory research innovation should include mitigating risk disparities across HUSVCs and well-resourced communities and improving infrastructure and community resilience. These actions require: (1) promoting transdisciplinary, use-inspired, and actionable research; and (2) co-developing, co-producing, and co-disseminating scientific research in close collaboration with end users, following principles of research and data justice (75). The latter was stressed in a Permitting Action Plan published by the US Office of Management and Budget, which stressed the importance of community-led mitigation measures as part of environmental review (55).

### Social, environmental, and economic development certification

Although specific methods to estimate risk disparities across communities or characterize sources of uncertainty are beyond the scope of this article, we recommend a certification scheme referred to as Social, Environmental, and Economic Development (SEED). The proposed SEED certification aims to train and focuses the attention of infrastructure project planners on the gaps that exist throughout each tier of the framework discussed above. The imperative for SEED certification arises from the urgency to rectify historical and systemic inequities perpetuated by existing policies and frameworks and to facilitate recognition of the interconnectedness of the social and technical dimensions of infrastructure. This interconnectedness is vital, as it recognizes that addressing inequities requires not only structural changes but also a transformative mindset that permeates both policy and practice. The proposed certification scheme embodies the commitment to not merely overlay existing structures but fundamentally reshape the landscape of infrastructure development. It offers a robust mechanism to hold projects accountable, ensuring that considerations of risk disparities, uncertainties, and community-specific adaptation plans are not token gestures but integral and concrete components of every infrastructure initiative. By prioritizing equity in funding mechanisms, incorporating diverse metrics, engaging communities, and fostering sectoral interdependencies, SEED certification sets a new standard for infrastructure projects—one that aligns with principles of justice, sustainability, and resilience.

**Figure 2.**
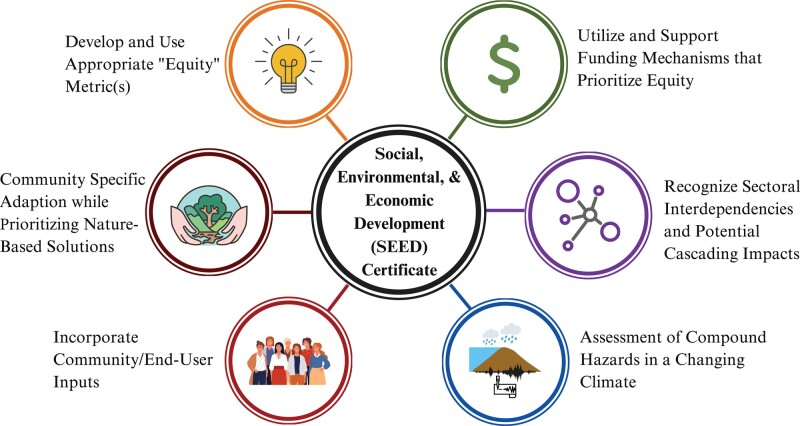
Proposed SEED certification scheme. SEED is a project certification scheme that minimizes risk disparities across HUSVCs and well-resourced communities.

The objective of SEED certification is to assess the extent to which a project considers risk disparities and the underlying uncertainties associated with planning for future hazards (Figure 2). Key elements include: (1) funding mechanisms that prioritize equity; (2) metrics that measure progress toward addressing various concerns related to equitable infrastructure, including appropriate “equitable risk” (76) metrics to measure progress; (3) community/end user inputs; (4) sectoral interdependencies and potential resulting cascading failures due to extreme events; (5) evolving impacts of compound extreme events under a warming climate; and (6) community-specific adaptation plans that prioritize nature-based infrastructure solutions to enhance both resilience and equity. In addition, certification requires the development of a clear and transparent process for community engagement and feedback, ensuring that community concerns and values are incorporated throughout a project's life cycle.

SEED certification is inspired by historical programs such as the US Green Building Council's Leadership in Energy and Environmental Design (LEED) certification and the US Environmental Protection Agency's EnergyStar program, as well as other low-emission certification schemes. Unlike the EnergyStar program, implementation of SEED does not stem from an act of Congress (i.e. the Clean Air Act) but rather would respond to a broader recognition of the social and environmental gaps related to justice, sustainability, and resilience as described above. Therefore, we recommend that further development of a certification scheme based on the framework presented herein, in addition to its implementation and enforcement, be led by an organization for which issues outlined by the framework are of increasing importance, particularly as they relate to infrastructure projects. A prime example would be the American Society of Civil Engineers (ASCE). Though ASCE recognized that social issues should be a concern for all civil engineers as far back as the early 1970s (77), it has until the present been unable to issue formal guidance or standards that incorporate said concerns into future infrastructure projects. As part of implementing the SEED certification process, the enforcing organization would initially develop each SEED category in terms of the specific concepts that will be scored, as well as determine and assign weights to the metrics that will be used to compute the scores for each category. It should also build capacity to not only develop a system by which future projects are assessed via the SEED certification scheme but also maintain a robust and transparent monitoring and reporting system to assess the outcomes and impacts of a project on the community and the environment throughout the life of the project.

SEED certification would provide a much-needed incentive to ensure that future infrastructure projects consider the issues and uncertainties related to equity, resilience, and sustainability that are addressed herein. While the concept of equity in infrastructure is not new, the novelty of SEED lies in the integration of restorative justice principles with a concrete certification scheme. SEED serves as a standardized and comprehensive tool to operationalize equity and offer a structured approach that goes beyond rhetoric, financial incentives, and federal law, while setting measurable standards for infrastructure projects. As we navigate a future marked by climate uncertainties and evolving risks, we envision SEED certification will stand as a beacon of progress, guiding various publics, as well as the scientific and engineering research communities, toward a more equitable, resilient, and sustainable infrastructure landscape. Further, it is a call to action, urging policymakers, practitioners, and communities to embrace a transformative approach that places a more holistic conception of justice at the core of infrastructure development. In the US, updates to agency definitions of “environmental justice” (to include “environmental risks” and “hazards” including those related to climate change and structural or systemic barriers), “accurate and clear climate change analysis” (to include “considering reasonably foreseeable effects of climate change on infrastructure investments”), and “cumulative impact” (to include capital investments affected by climate change) are indeed encouraging signs of progress (51, 53). Adoption of SEED certification would continue this momentum in terms of not only building infrastructure but also cultivating a future where every community, regardless of socioeconomic status, thrives in the face of change.

## Concluding remarks

The objective of equitable infrastructure should venture beyond simply adding an overlay to existing policies or a framework for incremental policy change. Instead, it should minimize risk disparities among HUSVCs and well-resourced communities (53, 76). This requires promoting not only innovative analytical but also exploratory scientific and engineering approaches to address both the root causes of HUSVC community formation and the evolving risks that result from or are exacerbated by policies that focus on procedural inclusion or the allocation of burdens (and, more recently, benefits) to communities identified via screening tools (78–80).

A comprehensive three-tiered framework is proposed to address the complex and dynamic interactions between infrastructure and equity through restorative justice (i.e. identifying and disrupting mechanisms that caused or contribute to inequities), incremental innovation (i.e. adjustments to policy, planning, and financial mechanisms), and exploratory innovation (i.e. synergistic advances in science and engineering). The first tier aims to recognize and reverse policy artifacts that perpetuate disparities and prioritizes restorative justice. The second tier entails policy, planning, and finance changes, including the use of analytic tools for racial and environmental equity, eliminating policy artifacts through rules, and implementing financial mechanisms to enhance climate adaptation, strengthen infrastructure resilience, and protect civil rights. The third tier focuses on addressing hindrances to exploratory innovation through engineering and scientific advances that target and limit risk disparities. While incremental and exploratory innovation can contribute to transformative adaptations, it is the focus on one at the expense of the other that places an organization or governance framework at risk of falling into innovation traps. We build our proposed framework from a grounding in restorative justice to acknowledge its unique and neglected ability to not only redress environmental harms but also focus on harms that are closely connected to past and present policy artifacts that contribute to inequities, specifically in the infrastructure domain. While distributive, procedural, and recognitional justice are crucial components of a comprehensive justice framework, the emphasis on restorative justice seeks to address historical and systemic inequities perpetuated by existing policies and practices. Restorative justice, with its focus on repairing harm and restoring relationships, is particularly suited to the complexities of infrastructure development where communities have faced prolonged disparities. While incremental and exploratory innovation are presented as distinct, they are not strictly sequential. In practice, these forms of innovation coexist and interact dynamically. Equitable infrastructure does not always require innovation in the sense of new technologies. Indeed, preventing explicit or implicit discrimination is a crucial aspect of creating equitable infrastructure and may not always involve groundbreaking innovations. At the same time, our framework acknowledges that innovation, incremental as well as exploratory, plays a vital role in addressing complex challenges and advancing solutions. Substantial incremental innovation (at a minimum), aided by and interacting with related exploratory research innovation over time, promises to achieve greater prevention and remedy of discrimination than the historical focus on cross-sectional identification of discrete programs and policies that yield disparate impact to one or more disadvantaged communities or protected classes.

A certification scheme, referred to as “Social, Environmental, and Economic Development (SEED)”, is proposed to further these objectives. Certification aims to focus the attention of infrastructure project planners on the gaps that exist throughout each tier of the framework. As the discourse on justice in infrastructure planning and development evolves, we recognize the need for ongoing dialogue and refinement of frameworks to incorporate diverse justice considerations.

## Data Availability

There are no data underlying this work.
